# Longitudinal ventilatory ratio monitoring for COVID-19: its potential in predicting severity and assessing treatment response

**DOI:** 10.1186/s13054-021-03768-2

**Published:** 2021-10-20

**Authors:** Natsuko Kaku, Yu Nakagama, Michinori Shirano, Sari Shinomiya, Kazuhiro Shimazu, Katsuaki Yamazaki, Yoshito Maehata, Ryo Morita, Yuko Nitahara, Hiromasa Yamamoto, Yasumitsu Mizobata, Yasutoshi Kido

**Affiliations:** 1 Department of Parasitology, Research Center for Infectious Disease Sciences, Graduate School of Medicine, Osaka City University, 1-4-3 Asahimachi, Abeno-ku, Osaka, 545-8585 Japan; 2 Department of Traumatology and Critical Care Medicine, Graduate School of Medicine, Osaka City University, 1-4-3 Asahimachi, Abeno-ku, Osaka, 545-8585 Japan; 3 Department of Infectious Diseases, Osaka City General Hospital, Osaka City Hospital Organization, 2-13-22 Miyakojima-hondori Miyakojima-ku, Osaka, 534-0021 Japan; 4 Department of Anesthesia and Intensive Care Medicine, Osaka City General Hospital, Osaka City Hospital Organization, 2-13-22 Miyakojima-hondori Miyakojima-ku, Osaka, 534-0021 Japan; 5 Department of Anesthesiology, National Cerebral and Cardiovascular Center Hospital, 6-1 Kishibe-Shimmachi, Suita, Osaka 564-8565 Japan

**To the Editor:**

We read with great interest the recent research article, published in *Critical Care*: “Longitudinal changes in compliance, oxygenation and ventilatory ratio in COVID-19 versus non-COVID-19 pulmonary acute respiratory distress syndrome”, by Beloncle and collaborators [1]. We agree with their conclusion that increase in ventilatory ratio (VR) during the first week of illness is characteristic to COVID-19 ARDS and reflects its uniqueness in pathophysiology. In addition, we herein wish to propose that VR in COVID-19 ARDS may serve as a potential bedside marker reflecting clinical severity and that its longitudinal monitoring may harbor prognostic value.

In our 28-day observational study including 39 patients with critically ill COVID-19 [2], longitudinal increase in VR values were associated with failure in discontinuing respiratory support (Fig. 1). Upon predicting failure, a VR threshold of 1.56 achieved the highest predictivity with a sensitivity of 0.667 and a specificity of 0.762 on day 5 of respiratory support. Of 21 patients with a VR value lower than 1.56 on day 5, 17 had successfully extubated within 28 days from respiratory support, suggesting that longitudinal VR monitoring could predict better outcome in COVID-19. Similar findings were obtained in another research applying VR changes from day 0 to 3 of respiratory support as a prognostic indicator [3]. Although statistically insignificant, Beloncle and collaborators have also shown an apparent trend towards better prognosis for a lower VR (Table S3; mortality in “VR < 2” versus “VR ≥ 2” were 15.5% versus 30%). It would be of great interest to validate our observations in their cohort as well, by assessing longitudinally the prognostic value of VR, if sufficient data were provided. The wide variety of VR values observed along the chronological course of COVID-19 ARDS, shown in Fig. 1D, may indicate the variable responses following therapeutic interventions. The expanding but yet investigatory list of therapeutics against COVID-19 warrants a deeper description of the therapeutic interventions received within the cohort.

**Fig. 1:**
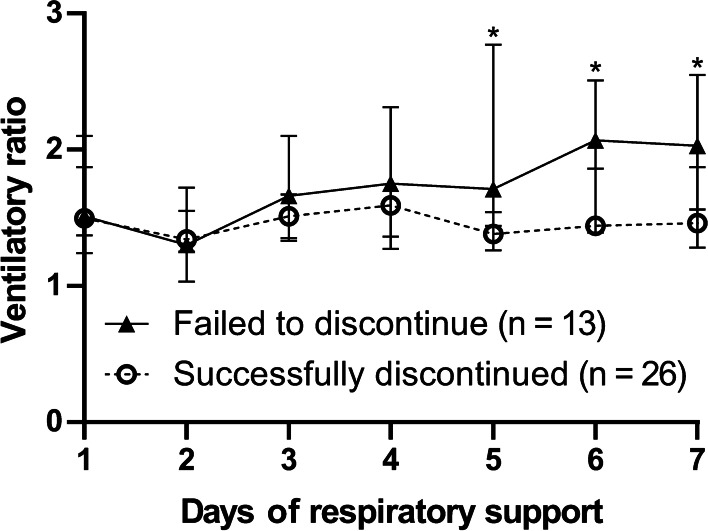
Longitudinal VR values after initiation of respiratory support. Longitudinal daily values of VR during the first week after initiation of respiratory support. The failed to discontinue group presented as the solid triangles and solid lines, and the successfully discontinued group presented as the opened circles and the dashed lines. Each marker placed on the median value, and each error bar meant the 95% confidential interval. Statistical analysis determined the differences between the failed to discontinue and successfully discontinued groups on each day by Mann–Whitney test. *p-value < 0.05

Elevation in VR, a surrogate marker of the increasing dead space fraction, is attributed to the progressive exudative damage affecting the alveoli, as well as the development of micro-embolism in the pulmonary circulation [4, 5], both known histopathological determinants of COVID-19 clinical severity. In addition to the here proposed prognostic value of VR monitoring in predicting natural history of COVID-19, future interest resides in whether longitudinal evaluation of VR may further reflect clinical response to treatment.

## Data Availability

The datasets analyzed in our study are available at https://www.medrxiv.org/content/10.1101/2021.07.20.21260754v1.supplementary-material.

## Abbreviations

COVID-19: Coronavirus disease of 2019
ARDS: Acute respiratory distress syndrome
VR: Ventilatory ratio
ROC: Receiver operating characteristic
