# Supplementary material for: Longitudinal ventilatory ratio monitoring for COVID-19: its potential in predicting severity and assessing treatment response
Source: Crit Care. 2021 Oct 20;25:366. doi: 10.1186/s13054-021-03768-2 (PMC8527974; doi:10.1186/s13054-021-03768-2)
Supplement: Related texts

# Reply: Authors’ response letter

**Authors:** François Beloncle, Antoine Studer, Valérie Seegers, Jean-Christophe Richard, Christophe Desprez, Nicolas Fage, Hamid Merdji, Bertrand Pavlovsky, Julie Helms, Sibylle Cunat, Satar Mortaza, Julien Demiselle, Laurent Brochard, AlainAlain Mercat, Ferhat Meziani

**Dear editor,**

We thank Drs Natsuko Kaku et al. for their interest in our study “Longitudinal changes in compliance, oxygenation and ventilatory ratio in COVID-19 versus non-COVID-19 pulmonary acute respiratory distress syndrome” that was recently published in Critical Care [1]. We also thank them for their valuable comment, in which they point out that an increase in Ventilatory Ratio (VR), a surrogate marker of an increase in dead space fraction [6], may allow to predict a poor outcome in patients with COVID-19 associated acute respiratory distress syndrome (ARDS).

We found in our cohort of 135 patients with COVID-19 associated ARDS that VR at day 1, day 3 and day 7 after intubation was higher in non-survivors than in survivors at day 28 (2.1 [1.7–2.4] vs 1.7 [1.5–2.0], *p* = 0.006; 2.4 [2.1–2.8] vs 1.9 [1.6–2.2], *p* = 0.003 and 3.2 [2.3–3.9] vs 2.3 [1.8–2.7], *p* = 0.001, respectively). Discrepancies between the two cohorts might be related to different time course evolutions which may be due to potential differences in non-invasive oxygenation strategies before intubation.

However, in line with the results reported by Drs. Natsuko Kaku et al., we found in patients with COVID-19 that the increase in VR from day 1 to day 7 tended to be higher in the non-survivors than in the survivors at day 28 (0.8 [0.3–1.8] vs 0.5 [0.1–0.9], *p* = 0.053). This tendency was not observed in the control patients with non-COVID-19 pulmonary ARDS. High VR are known to be associated with poor outcomes in patients with non-COVID-19 ARDS [6, 7]. Even if it has to be confirmed in larger cohorts, the potential specific prognostic value of the changes in VR over time in COVID-19 associated ARDS may be consistent with the distinct evolution of clinical features observed in this population during the first week of mechanical ventilation. As highlighted in our paper, this particular evolution may be in part due to different ventilatory strategies (in particular of positive end-expiratory pressure (PEEP) titration). We agree with Drs. Natsuko Kaku et al. that whether VR changes may help the clinicians to assess the efficacy of some therapeutics as PEEP levels or prone positioning is an interesting question which remains to be addressed.
